# Pelvic modelling and the comparison between plate position for double pelvic osteotomy using artificial cancellous bone and finite element analysis

**DOI:** 10.1186/s12917-018-1416-1

**Published:** 2018-03-20

**Authors:** William McCartney, Bryan MacDonald, Ciprian Andrei Ober, Rubén Lostado-Lorza, Fátima Somovilla Gómez

**Affiliations:** 1 NOAH, 38 Warrenhouse Road, Baldoyle, Dublin 13 Ireland; 2 Dublin City University, Glasnevin, Dublin 9 Ireland; 3 Department of Surgery, Faculty of Veterinary Medicine, University of Agricultural Sciences and Veterinary Medicine, Calea Manastur 3-5, Cluj-Napoca, Romania; 4 Department of Mechanical Engineering, University of La Rioja, Avda. de la Paz, 93, 26006 Logroño, La Rioja Spain

**Keywords:** Pelvic osteotomy, Hip dysplasia, Finite element analysis

## Abstract

**Background:**

Finite element analysis was used to compare fixation methods for double pelvic osteotomy (DPO). Using 3D scanning a stereolithography (stl) image was produced of a canine pelvis and this was subsequently refined in computer aided design (CAD). Using the CAD files, the images were imported in MSC Marc software to produce a working finite element (FE) model with 3 dimensional tetrahedral elements with linear shaped functions. The dimensions of a precontoured pelvic osteotomy plate with eight screws and a twisted seven screw straight plate were used to build the 2 fixations implants for the FE models. An equivalent load of 300 N was applied progressively on all FE models in order to facilitate its convergence. The load was applied in a distributed manner on the femur-hip joint contact area in order to simulate the actual behavior of the joint. The aim of the present study was to analyze the difference in stiffness and behavior under loading between a lateral vs ventral plate fixation, with unlocked screws and different gap scenarios, for stabilization of a pelvic osteotomy using finite element analysis**.**

**Results:**

From both configurations the maximum displacement of the ventral plate with 7 screws without gap had a value of 1.988 mm, while in the DPO plate had a maximum displacement of 2.191 mm. The load applied for each of the different configurations studied when a gap of 1° was considered and also when a condition of no gap was considered. The ventral plate was stiffer than the lateral plate when a gap was not present. When the gap was closed in the ventral plate, the stiffness increased until a point that remained constant.

**Conclusions:**

Ventral plate fixation can be as or more stiff as lateral plate fixation and provides flexible fixation. This behavior should reduce screw loosening. Using ventral plate fixation is recommended to reduce screw loosening or failure.

## Background

Triple pelvic osteotomy in dogs was first mentioned in the literature in 1969 [1]. Encouraging clinical and radiographic results were reported following triple pelvic osteotomy [2–6]. Thus, triple or double pelvic osteotomy has been used successfully to treat canine hip dysplasia [7, 8]. However, fixation complications are common in pelvic osteotomy repair and there has been little research into methods to reduce the rate of complications. Additional ventral fixation of the ilium decreased complications in a small group of dogs undergoing triple pelvic osteotomy and provided good stability for cadaveric oblique iliac osteotomy [9, 10]. In 2011, a study was undertaken to quantify the comparative benefit of using a ventral iliac plate in a double pelvic osteotomy model, and the effects of a gap in the fixation. Testing to compare the rigidity of the fixation methods were analyzed through the analysis of the degree of deformation under loading [11]. Anyway, complications rates for pelvic osteotomy can be very high with screw loosening at up to 62% being the most common problem [12–14]. Recommendations on screw selection and use have been made to reduce the incidence of screw loosening [15–17]. Locking screw use in pelvic osteotomy reduces the incidence of loosening [18–20]. Using either a triple or double pelvic osteotomy achieves similar acetabular ventroversion and have in effect the same result [11]. The purpose of this study was to analyze the difference in stiffness and behavior under loading between a lateral and a ventral plate for stabilization of a pelvic osteotomy using finite element analysis**.**

## Methods

Cadaver pelvis was used to scan and build pelvis model. Artificial bone was used in lab testing. Sourcing of materials was from a dog that died of natural causes and the artificial bones from Sawbones Company (Sweden). Using 3D scanning a stl image was produced of a canine pelvis (25 kg male Collie breed) and this was subsequently refined in CAD. Using the CAD files, the images were imported in MSC Marc software to produce a working finite element (FE) model with 3 dimensional tetrahedral elements with linear shaped functions. The dimensions of a precontoured pelvic osteotomy plate with eight 3 mm screws and a twisted seven 3 mm screw straight plate were used to build the 2 fixations implants for the FE models. The screws were fixed to behave as unlocked screws. Before the FE simulations were run validation bench tests were carried out using artificial cancellous bone blocks (Sawbones, Malmo). Osteotomy and plate fixation were set up in the 2 methods: 1. Ventral plate fixation 2. Lateral plate fixation. The load required to cause a deformation of set amount was recorded for 3 experiments for each plate position. This data was used to validate the FE simulation for all configurations.

**Fig. 1:**
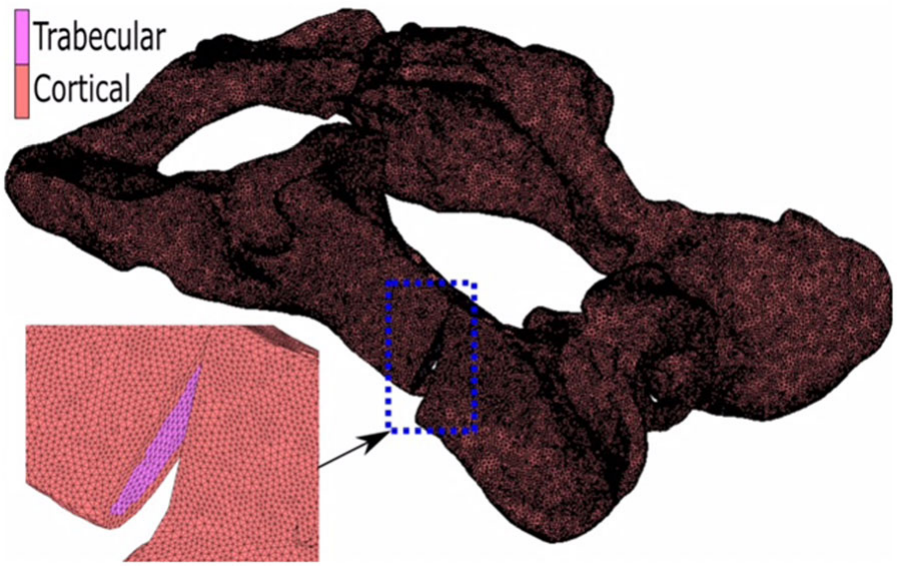
FE model of pelvis proposed in which is appreciated in the osteotomy in the cortical and cancellous bone

**Fig. 2:**
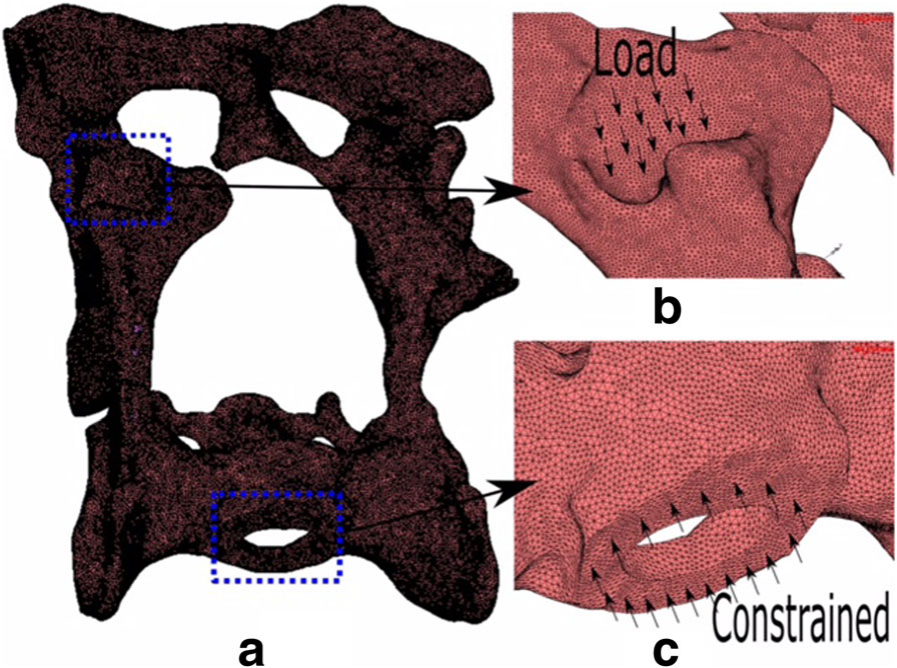
**a** Boundary conditions applied to the FE model pelvis: load (**b**) and constraints (**c**)

Finite Element (FE) models for each of the plate configurations studied were built to determine and verify the joint stiffness according to the applied load. Each of the FE models proposed that formed each configuration considered the osteotomised pelvis, the different plates and the screws. The geometric models used in the FEM discretization process of each of the parts (pelvis, plates and screws) were generated by measurements from actual samples of plates and artificial osteotomised pelvis. Due to the fact that the surface of the artificial osteotomised pelvis is very irregular; a 3d scanner was used to obtain its geometrical model. The remaining parts (plates and screws) were modeled directly in the FE MSC Marc software used. For all plate configurations studied, three dimensional FE models with 4-node isoparametric tetrahedral elements were used to mode the pelvis, plates and screws. An average element size of 0.6 mm was used for the cortical and cancellous bone, while for the plates and screws element sizes were for 0.8 mm and 0.9 mm respectively. An outer thin cortical bone and an inner cancellous bone were used to model the pelvis according to the literature. The material properties used for the cortical bone were E = 17,000 MPa and ν = 0.25, while for the cancellous values of E = 150 MPa and ν =0.3 were used. A titanium alloy with E = 107,000 MPa and ν = 0.34 was used for modeling of the plates and screws. The cortical and cancellous bone topography in the osteotomised area of the FE pelvis model was the same for all plate configurations (Fig. 1). A segment-to-segment method with a coefficient of friction of 0.1 was used for detecting mechanical contact between the plates and pelvis for all studied configurations. The mechanical contact between the screws and the osteotomised pelvis was defined via a glue contact between the matching nodes in both contact bodies. The same boundary conditions were applied in all finite element models in order to compare the different stiffness’s obtained. An equivalent load of 300 N was applied progressively on all FE models in order to facilitate its convergence. The load was applied in a distributed manner on the femur-hip joint contact area in order to simulate the actual behavior of the joint. The movement of the nodes that connected the pelvis to the column were constrained in order to immobilize the pelvis. Figure 2 shows a model of the pelvis which details the area where the load was applied and the area of the spinal column in which the constraint was applied.

Also, for all configurations studied, the value of the joint stiffness depending on the applied load was calculated according to the following equation:

$$ Stiffness=\frac{\  Applied\ Force}{Displacement\ Obtained} $$

**Fig. 3:**
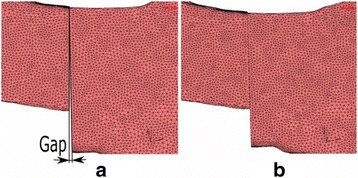
Detail of the gap considered (**a**) and not considered (**b**)

In this study, two types of different plate configurations were studied. The first configuration studied was a DPO plate with eight screws on the lateral ilium. The final configuration studied was a ventral plate and was fixed to the pelvis with seven screws. In addition, a small gap in the osteotomised pelvis area was taken into consideration for all configurations studied in order to determine their influence on the stiffness of the joints. This gap is defined as the separation corresponding to a one angular degree (1°) between the different parts in which the osteotomised pelvis is divided. Figure 3 shows a configuration of plate in which the gap has been considered (Fig. 3a) and another in which it has not been considered (Fig. 3b).

**Fig. 4:**
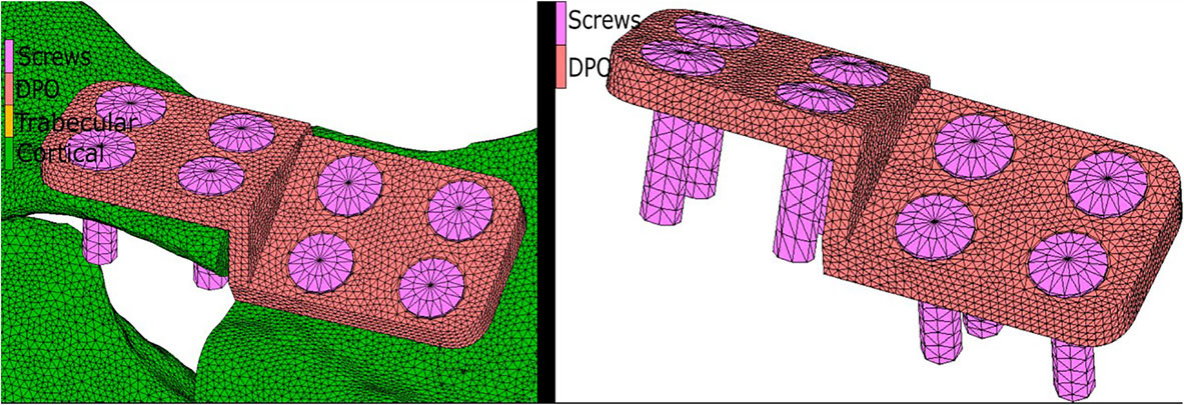
DPO plate mounted in the osteotomised pelvis. Detail of the DPO plate with the eight screws mounted

Figure 4a shows the DPO plate mounted on the osteotomised pelvis. Four screws were used on one side of the osteotomy while another four screws were used on the opposite side (see Fig. 4b). The dimensions of the plate were 15 × 3 mm and the diameter of the screws was 3 mm. In this configuration, all screws pass through the entire pelvis, and mechanical contact between the plate & cortical bone and between the plate & screws is created in order to completely define the contact in the plate. Contact between the bone fragments is defined in the elements close to the osteotomy in order to detect when the osteotomy gap is closed or open.

**Fig. 5:**
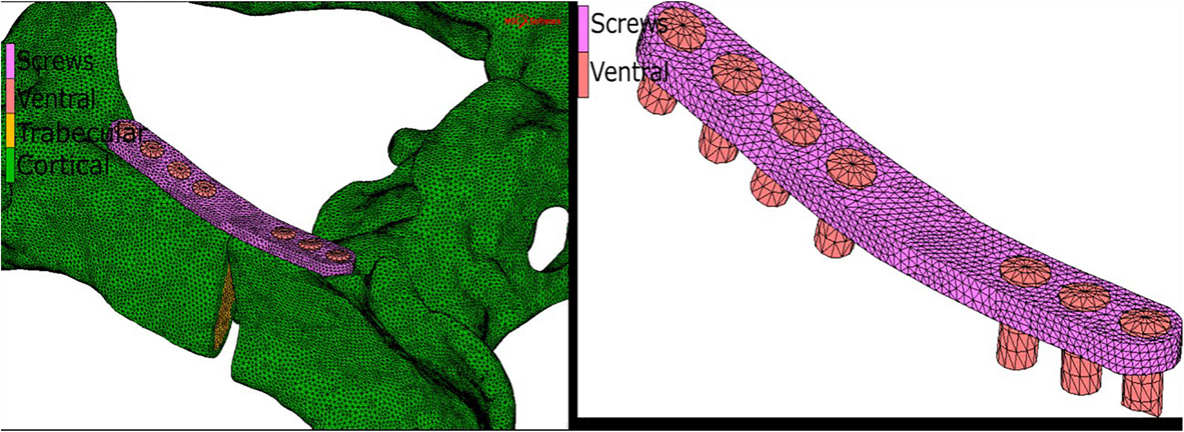
Ventral plate mounted in the osteotomised pelvis. Detail of the ventral plate with the seven screws mounted

**Fig. 6:**
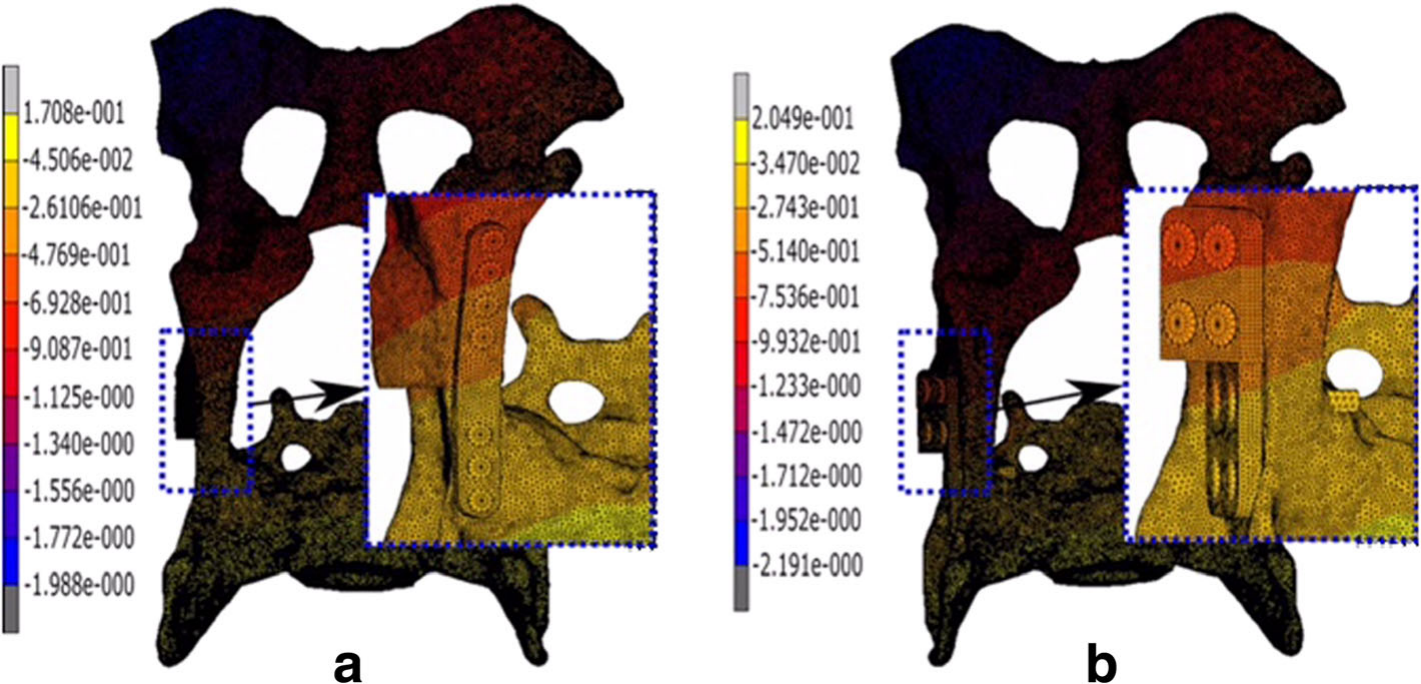
Maximum displacement obtained for the ventral plate of 7 screws without gap configuration (**a**) and for the DPO plate without gap configuration (**b**)

Figure 5a shows the ventral plate mounted on the osteotomised pelvis. Four screws were used on one side of the fracture while three screws were used on the opposite side (see Fig. 6b). The dimensions of the plate were 6 × 2 mm and the diameters of the screws were 3 mm. Similar to the previous configuration, contact between the bone fragments is defined in the elements close to the fracture site in order to detect when the gap is closed or open.

## Results

**Fig. 7:**
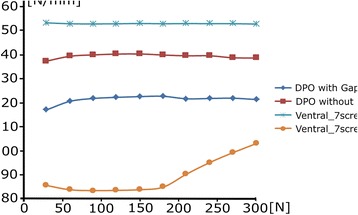
Variation of the stiffness obtained Vs. the load applied for each of the different configurations studied

The maximum displacement obtained from the osteotomised pelvis by applying a maximum load of 300 N when a ventral plate of 7 screws without gap (Fig. 6a) and a DPO plate without gap (Fig. 6) are illustrated. From both configurations it is observed that the maximum displacement of the ventral plate with 7 screws without gap has a value of 1.988 mm. while in the DPO plate has a maximum displacement of 2.191 mm. Figure 7 shows the variation of the stiffness obtained Vs. the load applied for each of the different configurations studied when a gap of 1° is considered and when a condition of no gap is considered.

## Discussion

The ventral ilium is the tension surface of the ilium and therefore is the natural choice for plating. During pelvic osteotomy the ventral ilium is exposed to allow retraction of the soft tissues medially, so there is no extensive extra tissue retraction to enable use of the ventral ilium for fixation. One of the authors (WMcC) routinely adds a ventral plate fixation to triple pelvic osteotomy fixation in giant breeds dogs, and this is a topic of further investigation.

Loss of fixation is the core issue in pelvic osteotomy complications. In non-locking plate fixation, the screws loosen in the cranial segment [15], whereas in locking plate fixation the loosening occurs in the caudal fragment. Although the number of reported cases repaired with locking plates is very low in the literature, the claim is that loosening is reduced overall by decreasing the stress on each individual screw. This is countered by the lower incidence of screw loosening in non-locking plates when the sacrum is included in the cranial screw fixation [15]. Another method to increase the overall stiffness of the fixation is to add a ventral plate [9]. Based on the results in this study, the addition of a ventral plate is theoretically justified and should lead to a reduced incidence of loss of fixation in any lateral plate fixation.

From this study it is observed that when a gap exists the fixation exhibits lower stiffness than the configuration that has no gap. Likewise, an increase in stiffness as the applied load increases is observed for ventral plate configurations. This variation in the stiffness is mainly due to the fact that the gap is closed at the ventral configuration as the load is applied, while in the DPO configurations type, this gap stays open as the load is applied. This means that DPO plates have a high stiffness in the area close to the fracture and prevent the gap from closing as the load increases. From these figures it can be deduced that the configuration of ventral plate of 7 screws without gap presents a higher stiffness than the configuration of DPO plate without gap. When the gap is closed in the ventral plate, the stiffness increases until a point that remains constant. The gap is important because depending on the dog size, the stiffness could take a low value or a high values and it is like a variable joint. The ventral plate has a dynamic aspect to its fixation. This effect does not occur on the pelvic osteotomy plate in which the stiffness keeps constant for all loads studied. Therefore, there is increased risk of screw loosening in the lateral plate.

The limitations of this study are that finite element verification using artificial bones may not reflect actual behavior bone loading in vivo.

## Conclusions

In conclusion, this study shows that ventral plate fixation can be as or more stiff as lateral plate fixation and provides flexible fixation. Using a ventral plate fixation is therefore recommended to reduce screw loosening or failure in double pelvic osteotomy to correct hip dysplasia in young dogs.

## Abbreviations

3D: Three dimensional
CAD: Computer-aided design
DPO: Double pelvic osteotomy
FE: Finite element
